# Editorial: Impact of physical activity on women’s health and quality of life: focusing on maternal health and pregnancy outcomes

**DOI:** 10.3389/fspor.2026.1787490

**Published:** 2026-02-23

**Authors:** Aziz ur Rehman Aziz, Jie Tan

**Affiliations:** 1 Key Laboratory for Early Diagnosis and Biotherapy of Malignant Tumors in Children and Women in Liaoning Province, Dalian Women and Children’s Medical Group, Dalian, Liaoning, China; 2 Department of Orthopaedics, Wuhan Fourth Hospital, Wuhan, Hubei, China

**Keywords:** clinical practice, disease prevention, physical activity, pregnancy outcomes, women's health

Pregnancy and the postpartum period are highly sensitive stages in a woman's life, characterized by significant physiological, metabolic, and musculoskeletal changes (1). Interventions during these phases can affect both maternal and neonatal outcomes, as well as their long-term health conditions (2). Among various factors, physical activity plays a crucial role, offering numerous immediate and long-term benefits for both maternal and child health (3). Physical activity can significantly reduce the risk of gestational diabetes, cesarian section, pregnancy-induced hypertension, pre-eclampsia, weight gain, intrauterine deaths, and preterm births (4–7). Exercise during pregnancy is associated with better mood, less depression, less anxiety and higher self-esteem. Moreover, exercise develops healthy immune systems in children whose mother have some moderate physical activity during pregnancy (8). Despite all these benefits, exercise is not often considered during routine maternal care. Moreover, concerns regarding safety, differences in individual responses, and a limited understanding of the underlying mechanisms limit the effective use of exercise recommendations in medical practice (9). This Research Topic aimed to investigate how physical activity and related physiological processes impact women's health and the quality of their lives during pregnancy and the postpartum period. The Research Topic includes articles related to coagulation dynamics, metabolic regulation, obstetric outcomes, and postpartum musculoskeletal recovery, covering more than just physical activity. The topic investigates the key benefits of physical activity within clinical and biological frameworks. These contributions support the idea that physical activity is a safe, adaptable, and clinically relevant strategy for optimizing maternal and neonatal health.

Li et al. examined thromboelastography (TEG) to evaluate the coagulation status of pregnant women. They revealed significant changes in TEG and traditional coagulation parameters as pregnancy progressed, reflecting a gradual shift towards a hypercoagulable state. This study highlights the importance of monitoring coagulation changes to ensure maternal and fetal health. However, these changes could be normal physiological adaptations for the development of a progressive hypercoagulable state of pregnancy rather than pathology. As exercise is a modulator of hemostasis and affects coagulation, moderate exercise can induce changes in TEG parameters and favor coagulation balance in pregnant women. However, a cautious approach should be adopted so that these patterns do not trigger any disease. Another article, not directly linked to physical activity, demonstrates positive long-term fertility and pregnancy results following surgical treatment of cesarean scar pregnancy.

Ma et al. revealed that post-treatment uterine adhesions are risk factors for failure to achieve pregnancy. No evidence is present that exercise can treat uterine adhesions. However, it may support uterine healing and fertility indirectly through improved circulation, metabolic health, and inflammation control. The study also points to the importance of thorough postpartum care, where physical rehabilitation and activity could be beneficial.

He et al. investigated metabolic regulation during pregnancy by recording daily energy expenditure of physical activity during the first, second, and third trimesters. They revealed that increased physical activity is associated with lower lipid concentrations and maternal age also has a significant effect on the metabolism of circulating lipids during pregnancy. These findings support the idea that physical activity can promote metabolic health during pregnancy and reduce complications related to dyslipidemia.

Similarly, direct evidence about the safety of physical activity during pregnancy is presented by Szablewska et al. They confirmed that physical activity (WHO recommended concentrations) before and during pregnancy does not have any negative impact on perinatal outcomes and neonatal condition in women who have given birth vaginally. Indeed, the study mentions some beneficial effects of physical activity on maternal health, such as cardiovascular function and thrombotic risk, promoting the need for appropriate exercise during pregnancy. However, further deep research is required for the confirmation of these mechanisms.

Lin et al. compared the differences in muscle thickness and contraction function of lumbo-pelvic-hip complex muscle between postpartum women with pelvic girdle pain (PGP) and asymptomatic controls using ultrasound imaging. Women with PGP presented reduced thickness, asymmetry, and diminished contractility of the lumbo-pelvic-hip complex muscle. These findings highlight the importance of targeted exercise-based rehabilitation to restore function and enhance quality of life after delivery.

In short, the articles in this research topic discuss how physical activity can affect physiological adaptation, metabolic regulation, and postpartum recovery. The articles support the idea that physical activity, according to WHO guidelines and customized to individual needs, is not only safe but also beneficial for maternal and child health. Thus, this collection successfully combines clinical, physiological, metabolic, and functional perspectives and claims that if physical activity is encouraged throughout pregnancy and the postpartum period, it can significantly improve the immediate and long-term health of women and their children. Future research should focus on revealing the mechanisms, refining dose-response relationships, and considering diverse populations to improve clinical and public health practices.

## References

[1] Bar A Moran R Mendelsohn-Cohen N Korem Kohanim Y Mayo A Toledano Y Pregnancy and postpartum dynamics revealed by millions of lab tests. Sci Adv. (2025) 11(13):eadr7922. 10.1126/sciadv.adr7922 40138427 PMC11939066

[2] Zavala E Rhodes M Christian P. Pregnancy interventions to improve birth outcomes: what are the effects on maternal outcomes? A scoping review. Int J Public Health. (2022) 67:1604620. 10.3389/ijph.2022.1604620 36405527 PMC9666362

[3] Majewska P Szablewska A. Associations between physical activity in pregnancy and maternal, perinatal, and neonatal parameters: a single-center prospective cohort study. J Clin Med. (2025) 14(7):2325. 10.3390/jcm14072325 40217775 PMC11989324

[4] Birsner ML Gyamfi-Bannerman C. Physical activity and exercise during pregnancy and the postpartum period: ACOG committee opinion, number 804*.* Obstet Gynecol. (2020) 135(4): p. e178–88. 10.1097/AOG.0000000000003772 32217980

[5] Ferrari N Joisten C. Impact of physical activity on course and outcome of pregnancy from pre- to postnatal. Eur J Clin Nutr. (2021) 75(12):1698–709. 10.1038/s41430-021-00904-7 33828239 PMC8636258

[6] Ribeiro MM Andrade A Nunes I. Physical exercise in pregnancy: benefits, risks and prescription. J Perinat Med. (2022) 50(1):4–17. 10.1515/jpm-2021-0315 34478617

[7] Muktabhant B Lawrie T Lumbiganon P Laopaiboon M. Diet or exercise, or both, for preventing excessive weight gain in pregnancy. Cochrane Database Syst Rev. (2015) 2015(6):Cd007145. 10.1002/14651858.CD007145.pub3 26068707 PMC9428894

[8] Moreno-Fernandez J Ochoa J Lopez-Frias M Diaz-Castro J. Impact of early nutrition, physical activity and sleep on the fetal programming of disease in the pregnancy: a narrative review. Nutrients. (2020) 12(12):3900. 10.3390/nu12123900 33419354 PMC7766505

[9] Laudańska-Krzemińska I Krzysztoszek J. Physical activity promotion among pregnancy – the role of physician from the women’s perspective. Front Public Health. (2024) 12:1335983. 10.3389/fpubh.2024.1335983 38487188 PMC10937457

